# Five suggestions for substantial NIH reforms

**DOI:** 10.7554/eLife.22471

**Published:** 2016-12-14

**Authors:** Michael Rosbash

**Affiliations:** 1 Department of Biology, National Center for Behavioral Genomics and the Howard Hughes Medical Institute, Brandeis University, Waltham, United States

**Keywords:** NIH, funding, science policy, careers in science, peer review, point of view

## Abstract

The National Institutes of Health needs to make radical changes to ensure that biomedical research continues to thrive in the United States.

The National Institutes of Health (NIH) has been the envy of biomedical scientists outside the United States for a long time. The NIH helped make the US a global magnet for talented researchers, fueled the growth of great research universities and is also indirectly responsible for the strength of the American biotech and pharmaceutical industries. However, the NIH and the US biomedical research enterprise now have a serious problem: there are too many researchers and institutions chasing after too few dollars. Some hope that consistent NIH budget growth can solve or at least seriously ameliorate this problem. However, it seems unlikely that increases in the NIH budget will suffice; major reforms are also needed.

The budget shortfall and other NIH problems are exacerbated by an organizational structure that has changed little over the past 50 years, and this is despite the revolutionary changes that have taken place in the life and biomedical sciences over this time. My goal in writing this article is to promote discussion that might catalyze serious changes in the way that the NIH operates.

## A brief history of the past 50 years

The NIH began in earnest in the late 1950s and now comprises 27 institutes and centers including, for example, the National Cancer Institute (NCI), the National Institute of Neurological Disorders and Stroke (NINDS), and the National Institute of Allergy and Infectious Diseases (NIAID). Although there have been some changes such as the addition of new institutes, the NIH is quite similar to when I began graduate school 50 years ago, in September 1966. This structure, with its focus on specific diseases, apparently began in part to facilitate political support from the US Congress.

In the early years of the NIH, it made sense that only a single institute, the National Institute of General Medical Sciences (NIGMS), was dedicated to basic science. Indeed, the importance of model organisms (fruit flies, nematodes, tetrahymena, mice and so on) to human health and disease was not well-appreciated by the medical community until the 1980s. This is when recombinant DNA technology enabled cloning, sequencing and genetic rescue experiments. Such work showed that all eukaryotes, indeed all life forms, use similar mechanisms. Although Darwin drew many of these same connections, the extent to which basic processes remain unchanged is mind-boggling. In my own field, work on fruit fly circadian rhythms identified genes and mechanisms that turned out to function almost identically in humans. There have been parallel changes in clinical medicine, with the approaches taken to different diseases being much more integrated today than they were in the past.

This sea change in knowledge forced a total reorganization of basic science departments in universities and medical schools. For example, many zoology and botany departments disappeared from research universities and were replaced by more integrated departments like cell and molecular biology. Ecology and evolution departments have undergone fusion or fission while incorporating new disciplines like population genetics. Many medical school departments of anatomy have closed or become cell biology departments, often with a substantial structural biology component. The key point is that these (and other) organizational changes make sense in light of what we have learned over the last 50 years. Moreover, all this change has been accompanied by an explosive increase in the size of the biomedical research community around the world.

No static organization can thrive in the face of such dramatic quantitative changes to its constituency and qualitative changes to its intellectual portfolio. Yet the disease-centric Balkanization of the NIH still dominates almost all aspects of funding: the appropriation of funds by Congress, the targeting by institutes of grant applications in particular directions, and the organization of peer review.

## The problems facing the biomedical research enterprise in the US

This project- and disease-based organization implies that we can steer research in a top-down manner. This is incorrect or at a minimum inefficient. Moreover, bottom-up investigator-initiated research, funded via the R01 mechanism, is the strength of the NIH system and also vital to combat disease and improve human health in the deepest, most effective way possible.

All of my younger colleagues and many senior colleagues now spend at least 1/3 of their time writing applications for research grants; this is time that should be spent doing research, teaching and training.

In addition (and this has been said many times), the peer review system does not and cannot function at current funding levels. Moreover, these funding levels are having multiple deleterious effects on the entire research community, especially on younger scientists. Although there is now much more emphasis at the NIH on first-time investigators, there is no strategy to address what happens when their first grant runs out and they have to compete with everyone else, including the most established researchers, for the small amount of available funding. This is a recipe for disaster for the future of US science.

All of my younger colleagues and many senior colleagues now spend at least 1/3 of their time writing applications for research grants; this is time that should be spent doing research, teaching and training. The latter activity includes inspiring the next generation of scientists, which is hard to do when you are depressed and desperate.

An additional pernicious consequence of the current project- and disease-based system, as well as poor funding climate, is that some researchers claim that their work is relevant to disease when it is not – at least not yet. Although "tell the complete truth" is the mantra of doing research and the bedrock principle of mentoring students and postdocs, some scientists end up stretching the truth. To make matters worse, this is happening at a time when our community is under increasing and legitimate pressure to make sure that results are reproducible. I am not referring here to misconduct or to problems with data but to highly questionable claims of relevance to disease in publications. I am also referring to disingenuous statements about intended projects, experiments and disease-relevance in grant applications.

Changes to the NIH over the past 15 years have been much too modest to solve the problems that currently exist, and I am not alone in thinking that they require a robust debate to find solutions that the whole community will support (see, for example, Alberts et al., 2014; Alberts et al., 2015; Berg, 2012; Bourne, 2013a, 2013b; Kimble et al., 2015; McDowell et al., 2014; Rosbash, 2010; Yamamoto et al., 2016; Zoghbi, 2013). Here I propose five specific reforms.

## 1. Increase support for bottom-up basic science

The NIH pays substantial attention to disease-based Requests for Applications (RFAs), translational research and special initiatives (such as the Cancer Moonshot and the Brain Initiative). There is internal as well as external pressure to maintain and even expand these parts of the NIH research portfolio. Yet many researchers believe that human health writ large will benefit in the long run from less top-down direction. When the time is right for translational research, applications will emerge naturally from the bottom-up R01 mechanism that is used to fund most investigator-initiated applications. Top-down translational research and contract work should be left to biotech, big pharma, philanthropy and other non-federal funding sources.

To develop more robust therapies against disease, it is essential that we play the long game and better understand how cells and neurons normally function.

An example of a poorly conceived top-down initiative, in my opinion, is the money recently allocated to a set of hastily constructed RFAs related to Alzheimer's disease by the National Institute of Aging. Although Congress mandated this increased Alzheimer's funding, it is not clear to me that the aging-Alzheimer's research community is in a position to make good use of such a windfall. Recent breakthroughs in cancer immunotherapy, on the other hand, are an example of translational research that builds on decades of basic science funding – admittedly during a more generous era.

Although disease-oriented initiatives are easy to explain, promote and defend, the NIH and the scientific community have not worked hard enough to educate Congress and the public about the importance of basic science and the R01 mechanism. Moreover and despite enormous progress, we still have only a rudimentary understanding of many areas of biology, particularly the brain. To develop more robust therapies against disease, it is essential that we play the long game and better understand how cells and neurons normally function.

## 2. Move most support for basic science to a single institute

NIGMS is presently the only institute with basic science as its core mission. Although other institutes also fund basic science, there is a lack of coherence, and some institutes often conflate disease focus and basic science goals. I suggest that most support for basic science be shifted to NIGMS (or to a new "super-institute" focused on top-flight basic science). To this end, each institute should transfer a fraction of its budget to NIGMS (or the new super-institute) over the next 10 years. As an extreme example, if this budget distribution were to change by 5% a year, in a decade 50% of total NIH dollars would fund basic research through a single institute. Independent of the amount and the mechanism, the NIH needs to increase its support for basic science and ensure that more of the grant applications in basic science are in direct competition with each other.

## 3. Invest more in people and less in projects, but with limits

Decisions about funding much of basic science should focus more on the track record of investigators and less on the nature or relevance of the projects being proposed. (I also prefer a path to funding with no evaluation of project.) For newly independent investigators, this would mean relying on the research they did during their graduate and postdoc years. Outstanding accomplishments in two different environments constitute a first-rate track record for a new investigator.

An excellent track record from an established investigator would justify the following statement: "Here's what I've done for the past five years. Based on these accomplishments, please give me money to continue my current work. I'm not quite sure how it will pan out, nor am I certain what I'll accomplish over the next five years. I also tend to switch horses in midstream if our data take us in an unanticipated direction. In any case, I'll report back in five years, and you can judge whether my progress has been sufficient, qualitatively as well as quantitatively, to merit funding once again and at what level." In this way, grant writing would not only take less time but also become a less important skill. In the current system, the "brief" – as one scientist called his carefully crafted grant – often overshadows accomplishment or even intent.

Versions of track record-centric funding are already in place at some institutes (such as the R35 mechanism at NINDS and NIGMS), and a similar policy could be applied NIH-wide to fund many more outstanding researchers at the peak of their careers. I suggest that they could receive between $500k and $750k per year over a period of seven years. This is similar to the maximum support that an individual PI can receive from NIGMS ($750k per year) and to the prestigious Pioneer Grant program ($700k per year), which funds a small number of creative out-of-the-box applications.

I would go further and suggest that researchers receiving substantial funding from any NIH track-record-focused program should not be allowed to receive additional funding from any other NIH source. Although exceptions could be made, perhaps for work that is unusually expensive, people with very high levels of NIH funding should also be cut back to something like $750k per year.

It follows that the current practice of completely open competition at the NIH without regard to research support from private sources also deserves serious discussion. For example, more than 80% of the 320 Howard Hughes Medical Institute (HHMI) investigators currently have NIH grants. Their scientific mettle makes them formidable competition for researchers who rely solely on NIH funding.

The above recommendations would reduce the number of established, well-funded PIs in the competitive system, which would allow study sections to focus on individuals whose applications require more attention. In addition, early-career scientists would be competing against fewer highly productive senior scientists head-to-head.

I should stress that I am not proposing that NIH funding only go to the most established people and programs. On the contrary, the US requires a broad research base, for educational as well as research purposes. How should NIH funding be distributed between these groups? There are a number of related funding issues that also need addressing. For example, which mechanisms and training programs should be continued and why? Do we need to make adjustments based on geographical distribution? If so, to what extent? For all of these reasons, the research community must engage in a serious debate about the NIH and how it allocates its money, rather than sticking with the status quo. This debate should also cover the distribution of funds between the different NIH institutes, as well as the distribution between intramural research at the NIH's own laboratories and extramural research at universities, medical schools and research institutes.

## 4. Increase participation by established scientists in NIH Study Sections

The NIH relies on Study Sections to peer review the grant applications it receives. Although Study Sections have an almost impossible job given current funding levels, they still need serious improvement. For example, some very established scientists have never served on a Study Section, and many serve only rarely. I suggest that receiving NIH support should obligate a PI to serve on Study Section at some frequency, for example no less than once every three years. And Study Sections should be required to have a minimum percentage of established scientists at every meeting, for example at least three members with 20 years or more of NIH funding. Experience and wisdom are invaluable.

## 5. Revisit financial relationships between universities and the NIH

Indirect costs need to be controlled and perhaps substantially reduced. I am not sufficiently expert to offer specific recommendations, but indirect costs should be studied by an independent panel, e.g., one that is not dominated by deans or former deans of medical schools. Relevant to indirect costs are the "kickback" schemes that funnel indirect costs back to research laboratories at some institutions. Although fungible private funds make this practice hard to control, it should be forbidden.

Some universities and research organizations rely on grants to pay a large fraction of PI salaries. I recommend that at least 50% of a PI salary should be covered by the host institution. To achieve this end within a decade for example, a cap should be introduced on the percentage of PI salary support from the NIH, beginning with 95% next year and ending with 50% 10 years from now. Importantly, PIs with less than this fraction of institutional salary support should not be allowed to apply for NIH grants. Some policy of this nature will not only lead to a welcome reduction in the number of grant applications but also encourage institutions and philanthropy to contribute more to PI salary support.

I recognize that a good argument can be made to the contrary. If a PI works 100% on a grant, why can't his/her salary be 100% covered by that same grant? However, many such PIs do not spend 100% time on that grant, e.g., they collaborate on other projects or have some teaching obligations. In addition, this suggestion reflects a choice: in my view, NIH funds that cover these investigators would be better spent supporting work at institutions with more substantial PI salary support.

## In closing and independent of my specific suggestions

My generation experienced a golden age in the US. We lived free of world wars and benefited – personally and professionally – from a remarkable economic and intellectual expansion. The genetic code was being cracked as I was taking my first biology course as an 18 year-old chemistry student at Caltech in 1962; every week Professor James Bonner would fill in a table on the blackboard with a new triplet or two. The advent of recombinant DNA revolutionized my research program shortly after I established my laboratory at Brandeis in the mid-1970s. Importantly, research funding was relatively easy to obtain throughout most of my career. As a consequence, I owe an enormous debt to the NIH and to the US taxpayer for giving me this wonderful career. (I owe a similar debt to HHMI, but that is another story…)

US research universities have also benefited from NIH largesse, and many of them took advantage of indirect cost rules to increase faculty size and research infrastructure (mostly new buildings) at NIH expense (Alberts, 2010). Although these institutions and arguably the country benefited from such entrepreneurial activity in good times, the current situation bears some resemblance to the recent financial crisis; too much speculation now risks inflicting serious damage to the US research enterprise.

Indeed, the preeminent position of the US in life science research and the health of its biomedical research enterprise are not sustainable without bold reforms to maintain a robust NIH and research community. A failure to act will threaten many of the nation's great research universities as well as its biotech and pharma industries.

There is also a more subtle need for NIH reforms: they are necessary to counter the increasingly "business as usual" or "I have to get mine" mindset that results from a damaged system. In science and medicine as well as in politics, the compromising of truth ("truthiness" in the word of Stephen Colbert) is both symptom and disease. We should be vigilant to keep suspicions about evidence and fact, as well as corruption and cynicism, from poisoning our community and the system we leave behind. This is not only important pragmatically but also for ethical and educational reasons.
